# Proteomic profiling of bronchoalveolar lavage following human segmental endotoxin challenge—a potential exacerbation model

**DOI:** 10.1038/s41598-026-39528-x

**Published:** 2026-02-12

**Authors:** Christina Gress, Meike Müller, Jens M. Hohlfeld

**Affiliations:** 1 Fraunhofer Institute for Toxicology and Experimental Medicine ITEM, Hannover, Germany; 2 German Center for Lung Research (DZL-BREATH), Hannover, Germany; 3 Department of Respiratory Medicine and Infectious Disease, Hannover Medical School, Hannover, Germany; 4 Fraunhofer Institute for Toxicology and Experimental Medicine ITEM, Clinical Airway Research, 30625 Hannover, Germany

**Keywords:** Biomarkers, Diseases, Immunology, Medical research

## Abstract

Segmental lipopolysaccharide (LPS) challenge is a well-established method to induce airway inflammation in human lungs. Bronchoalveolar lavage (BAL) is typically used to capture molecular and cellular changes, allowing investigation of pulmonary inflammation and assessing drug efficacy. This study aimed to describe the proteomic profile of human BAL in the segmental LPS challenge model. Pre-challenge BAL was collected from ten healthy non-smoking participants, followed by segmental instillation of LPS (40 EU/kg) and saline in the contralateral lung as control. After 24 h, BAL was sampled from the challenged lung segments. Using the SomaScan platform, 1,500 proteins were analysed. As expected, no significant differences were found between pre-challenge and saline-challenged BAL. After LPS compared to saline challenge, 599 proteins such as IL-6, IL-8, MPO, MMP9, CRP, VWF, G-CSF and Eotaxin, were significantly upregulated and 4 proteins were downregulated in BAL. The most significant biological processes associated with upregulated proteins were “*immune system process*”, “*response to external stimulus*” and “*response to chemical*”. As expected, the LPS challenge triggered a strong pro-inflammatory response including proteome changes seen in acute exacerbations (AEs). Overall, this study provides a comprehensive analysis of the proteomic profile in BAL following LPS challenge and its similarities with AEs, contributing to a deeper understanding of this model.

**Supplementary Information:**

The online version contains supplementary material available at 10.1038/s41598-026-39528-x.

## Introduction

Chronic obstructive pulmonary disease (COPD) is a widespread, multifactorial airway disease characterized by persistent respiratory symptoms, airflow obstruction, and progressive, irreversible damage to the lungs, often resulting from a combination of chronic inflammation, oxidative stress, and structural changes in both the small airways and lung parenchyma^1^. COPD is primarily caused by long-term exposure to tobacco smoke or environmental pollutants, with genetic factors and respiratory infections also contributing to chronic lung inflammation and damage. Acute exacerbations of COPD (AECOPD) are defined as sudden worsening of lung function and symptoms, mainly caused by inflammatory responses to bacterial or viral infections^1^. The primary aims of AECOPD treatment are to alleviate symptoms, to reduce the severity and duration of exacerbations, and to prevent rapid loss of lung function by using, for example, bronchodilators, corticosteroids or antibiotics^1^. However, treatment options are very limited and exacerbations remain a major disease burden in COPD. Therefore, better strategies for prediction, early detection, and prevention of AECOPD as well as new specific anti-inflammatory and immune-modulating drugs are urgently needed.

The translation of findings from preclinical studies to humans is a crucial step in early clinical drug development. Preclinical studies, often conducted in vitro or in animal models, provide essential initial data on the efficacy, toxicity, and pharmacokinetics of new drug candidates. However, significant genetic, anatomical and physiological differences between animal and human lungs restrict translation, emphasizing the importance of early human proof-of-concept studies^2,3^. The mentioned differences can result in variations in drug absorption, distribution, metabolism, and excretion, leading to discrepancies in drug efficacy and safety profiles observed in preclinical studies versus clinical trials. The translation of findings from preclinical studies to humans usually involves initial studies with healthy volunteers. However, one significant limitation of non-diseased organs in healthy volunteers is the absence of specific pathological features including pathway activation, relevant cell migration, and inflammation^3^. Human challenge models with e.g., endotoxin, allergen or infectious compounds offer the possibility of inducing specific inflammatory responses in healthy volunteers that resemble aspects of diseases such as AECOPD and are therefore valuable for testing the efficacy of investigational new drugs or vaccines^4–6^.

Segmental lung challenge with lipopolysaccharide (LPS), an endotoxin from the outer membrane of gram-negative bacteria, is a well-established method to induce transient airway inflammation in healthy participants^6^. This model involves the direct instillation of LPS into a specific lung segment via bronchoscopy, which leads to localized inflammation. The inflammatory response induced by LPS can be thoroughly assessed through bronchoalveolar lavage (BAL) fluid collected from the challenged lung segment. Notably, there is a significant infiltration of inflammatory cells into the lung following LPS challenge, which is dominated by neutrophil granulocytes^7^. Therefore, the LPS challenge model mimics some aspects of COPD and exacerbations thereof^8,9^. In line, transcriptomic analysis of BAL-derived cells from the segmental LPS challenge model revealed overlapping features with respiratory diseases in general and infection-triggered respiratory insults such as AECOPD or community-acquired pneumonia in particular^10^. Furthermore, pro-inflammatory cytokines such as interleukin 6 (IL-6), IL-8, albumin, and myeloperoxidase (MPO) are increased in BAL and sputum after LPS challenge^7^. However, to our knowledge, there is no comprehensive analysis of the human proteome in the LPS challenge model and its overlap with AECOPD to date. While investigation of individual selected proteins allows only limited interpretation, proteomic profiling offers the opportunity to identify new biomarkers, protein-interactions and signalling pathways that are involved in infection-triggered inflammatory responses. Such insights are crucial for understanding the molecular basis of airway inflammation in general and endotoxin-induced inflammation in particular.

The aim of this study was to characterize the proteomic profile in BAL fluid following LPS challenge and to contextualize the data with AECOPD processes.

## Materials and methods

### Study design and ethics approval statement

Healthy participants underwent bronchoscopy with segmental LPS challenge. Pre-challenge baseline BAL was collected followed by segmental instillation of LPS and saline in the contralateral lung as control. After 24 h, BAL was sampled from the challenged lung segments. Participant demographics and further details of the study design, including clinical monitoring procedures and outcomes, have been reported previously^11^. Here, a total of 1,500 proteins were assayed in cell-free BAL supernatants using the SomaLogic SomaScan platform. Protein data were analyzed and compared with a systematic review by Chen et al.^12^, where selected blood biomarkers of exacerbations in COPD were elaborated. The protocol was approved by the independent Ethics Committee of the Hannover Medical School (Approval No. 7193) and registered at clinicaltrials.gov (NCT03044327, 07/02/2017) prior to study start. The study was conducted at the Fraunhofer Institute for Toxicology and Experimental Medicine, Hannover, Germany in accordance with the Declaration of Helsinki and the International Council for Harmonisation (ICH) Harmonised Tripartite Guideline for Good Clinical Practice (GCP).

### Participants and consent to participate

Ten healthy, non-smoking participants (7 males and 3 females, smoking history < 1 pack-year, mean age: 38 ± 10 years) with a body mass index (BMI) of 18.5–29.9 kg/m^2^, and normal lung function (forced expiratory volume in 1 s (FEV_1_): 101 ± 14% predicted) were included. Written informed consent was obtained from all participants after they were fully informed about all trial-related aspects before any study-related procedures.

### Sample collection

Bronchoscopies were performed according to the guidelines for investigative bronchoscopies^13,14^. Participants underwent a first bronchoscopy to collect pre-challenge baseline BAL from a segment of the left lower lobe using 100 mL (5 aliquots of 20 mL) of pre-warmed saline, following segmental challenge with LPS (40 endotoxin units per kg body weight diluted in 10 mL of saline; Endotoxin from E. coli Type O113; List Biological Laboratories Inc., Campbell, California, USA) in the medial segment of the middle lobe and 10 ml saline (0.9%) in the medial segment of the lingula lobe as control. A second bronchoscopy was performed twenty-four hours later for collection of BAL (100 mL) from the saline- and LPS-challenged lung segments.

### Processing and analysis of BAL samples

BAL samples were filtered (100 μm, BD Biosciences, Heidelberg, Germany) and centrifuged (300 g, 10 min, 4 °C). Cell pellet was resuspended in Dulbecco’s phosphate-buffered saline (DPBS), and total cell counts were determined by light microscopy using trypan blue staining and a Neubauer chamber. Absolute cell numbers were normalised to BAL volume recovery [10^6^/mL]. Differential cell counts were determined by counting 800 cells microscopically on cytospins stained with Diff quick (RAL Diagnostics, Martillac, France). Cell-free BAL supernatants were stored at -80 °C until analysis. Total protein concentration in BAL supernatants were determined using the colorimetric Pierce™ 660 nm protein assay (Thermo Fisher Scientific, Rockford, Illinois, USA). Based on total protein content, samples were diluted with saline to a protein concentration of 50 µg/mL. Subsequently, 1,500 proteins were analysed by SomaLogic using the SomaScan assay platform (complete list of proteins is given in supplementary Table 1)^15,16^.

## Statistical analysis

SomaScan data underwent quality control and transformation based on bioinformatic standards^17^. The lower limit of detection (LLOD) was defined as mean of eight blank samples plus three standard deviations (SD) of the blank. For the upper limit of detection (ULOD) a cutoff signal of > 100,000 was determined, as dilution linearity was not given for values above this cutoff (supplementary Fig. 1). Proteins with signal values below LLOD or above ULOD were excluded from further analysis. Signal values were then normalised to the original protein concentration of the respective sample. For analysis of expression differences in BAL following LPS compared to saline challenge, paired t-tests were performed, and p-values were adjusted (adj.) for multiple testing using the Benjamini-Hochberg procedure, which controls false discovery rate (FDR). Significance level was set to 0.05. Differential expressed proteins were defined by the criteria adj. p-value ≤ 0.05, and Log2 fold change (|Log2FC|) ≥ 0.58. Spearman correlations were applied to determine correlation of data obtained by the SomaLogic SomaScan assay compared to previously published data obtained by measurement of IL-6, IL-8, MPO and SP-D using the Meso Scale Discovery assay^7^ (data presented in Holz et al.^7^ originate from the same study, thereby allowing for correlation analysis).

### Data visualization

Data were visualized using GraphPad Prism 9.0.1. Principal component analysis (PCA)-Plots were created using SomaLogic DataDelve™ Statistics^18^. Gene set enrichment analysis was performed with upregulated differentially expressed proteins using *g: Profiler* (Version 2019) to determine enriched biological processes (GOTERM_BP_DIRECT)^19^. Adj. p-values were calculated for multiple testing by *g: Profiler* using the Benjamini-Hochberg procedure, which controls FDR. Protein network was visualized by using the STRING database (https://string-db.org/)^20^. STRING protein-interactions were filtered based on a confidence score threshold of 0.9 (highest confidence). Hierarchical protein clustering of the top 100 differentially expressed proteins with the highest Log2FC after LPS compared to saline challenge was performed with ClustVis using default settings (https://biit.cs.ut.ee/clustvis/)^21^.

## Results

**Fig. 1:**
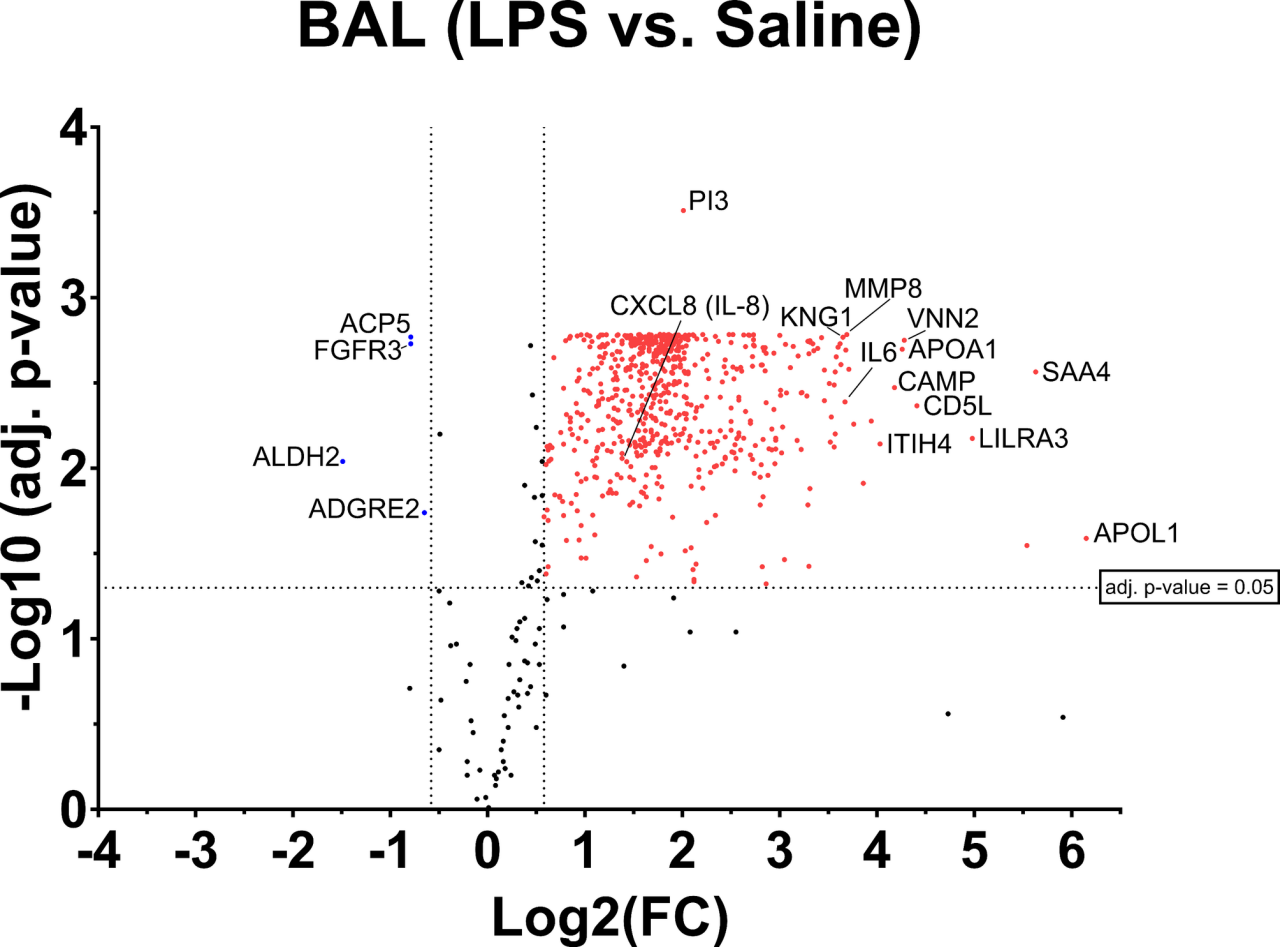
Differentially expressed proteins in BAL following LPS challenge compared with saline control challenge. Differentially expressed proteins are highlighted in red for upregulated proteins (adj. p-value ≤ 0.05, log2(FC) ≥ 0.58) and in blue for downregulated proteins (adj. p-value ≤ 0.05, log2(FC) ≤ − 0.58). Abbreviations: ACP5 = Tartrate-resistant acid phosphatase type 5, ADGRE2 = Adhesion G proteincoupled receptor E2, Adj. = adjusted, ALDH2 = Aldehyde dehydrogenase 2 (mitochondrial), APOA1 = Apolipoprotein A-I, APOL1 = Apolipoprotein L1, BAL = bronchoalveolar lavage, CAMP = Cathelicidin antimicrobial peptide, CD5L = CD5 antigen-like, CXCL8 = C-X-C Motif Chemokine Ligand 8, FGFR3 = Fibroblast Growth Factor Receptor 3, IL = Interleukin, ITIH4 = Inter-alpha-trypsin inhibitor heavy chain H4, KNG1 = Kininogen 1, LILRA3 = Leukocyte immunoglobulin-like receptor subfamily A member 3, Log2(FC) = Log2 fold change, LPS = lipopolysaccharide, MMP8 = Matrix Metallopeptidase 8, PI3 = Elafin, SAA4 = Serum amyloid A-4 protein, VNN2 = Vanin 2.

As expected, LPS challenge induced recruitment of high numbers of inflammatory cells, predominantly neutrophils, which was not observed following saline challenge (supplementary Table 2). After challenge with LPS compared with saline, four proteins (ALDH2, FGFR3, ACP5, ADGRE2) were downregulated and 599 proteins were significantly upregulated in BAL (Table 1; Fig. 1, supplementary Table 3). As expected, upregulated proteins were predominantly pro-inflammatory mediators, including IL-6, IL-8, MPO, LTB4, MMP9, VWF, SELL or G-CSF.

In accordance with the differential protein expression analysis, principal component analysis as well as a heatmap display showing the top 100 proteins with highest Log2FC clearly separated BAL samples following LPS challenge from those collected at pre-challenge baseline or after saline control challenge (supplementary Fig. 2, supplementary Fig. 3). As expected, the proteomic profile in BAL collected at pre-challenge baseline is similar to that in BAL collected after saline challenge, resulting in a single cluster of these samples (supplementary Fig. 2). Notably, participant 7 exhibited a more pronounced and participant 10 a weaker immune response to LPS compared to other volunteers (supplementary Fig. 3). As neither participant showed any abnormalities during the procedure (e.g., adverse events or signs of acute infection with elevated leukocyte counts), their data were not excluded from further analysis. Hierarchical clustering using the top 100 differentially expressed proteins did not reveal distinct protein subgroups (supplementary Fig. 3).

**Fig. 2:**
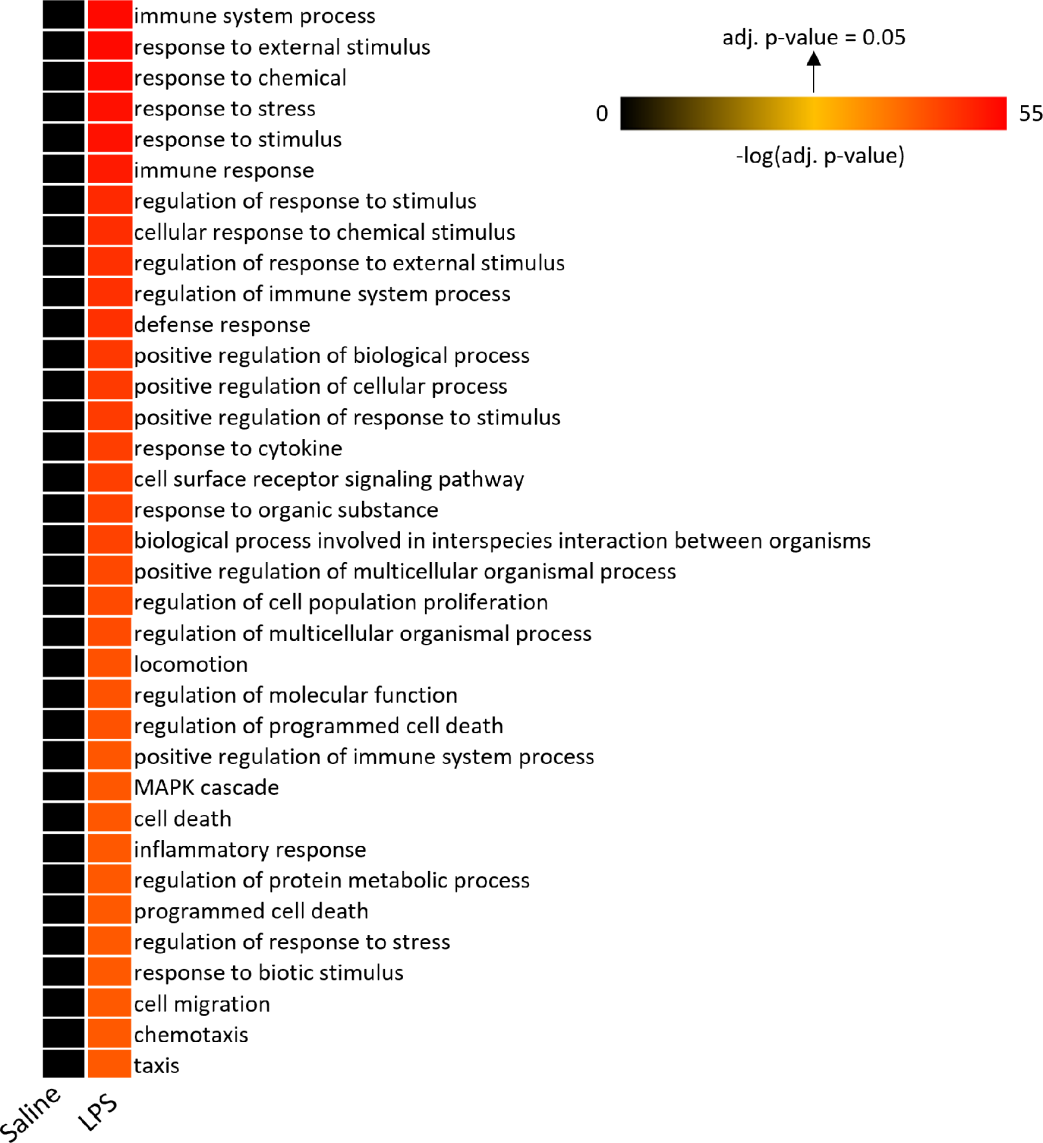
Enriched biological processes that are related to upregulated proteins (adj. p-value ≤ 0.05, log2(FC) ≥ 0.58) in BAL following LPS challenge compared with saline challenge. The 35 most significant results are depicted (range of adj. pvalues: 3.4e-28 to 6.1e-53). Abbreviations: Adj. = adjusted, BAL = bronchoalveolar lavage, Log2(FC) = Log2 fold change, LPS = lipopolysaccharide

Significant upregulated proteins (adj. p-value ≤ 0.05, Log2FC ≥ 0.58) were used as input for gene set enrichment analysis using *g: Profiler*^19^. The three most significantly enriched biological processes were “*immune system process*”, “*response to external stimulus*” and “*response to chemical*” (Fig. 2, supplementary Table 4). Other significantly increased biological processes were related to, for example, apoptotic or migration processes (Fig. 2). Due to the small number of downregulated proteins in BAL in the LPS challenge model, no gene set enrichment analysis was performed for this group.

**Fig. 3:**
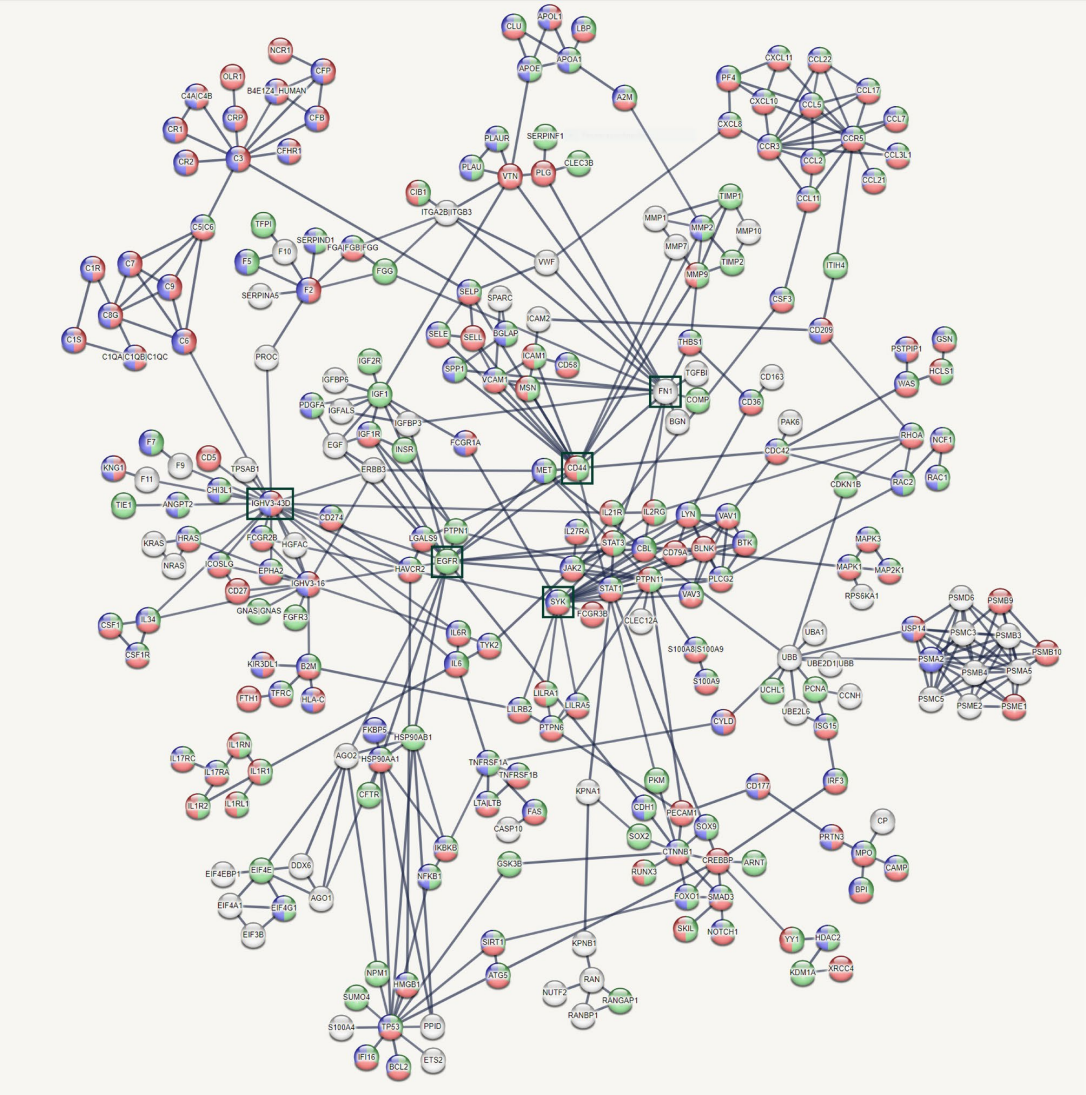
Network analysis, depicting protein interaction clusters of significantly upregulated proteins (adj. p-value ≤ 0.05, log2(FC) ≥ 0.58) in BAL following LPS challenge compared with saline challenge. Proteins that are related to the three most significant enriched biological processes „immune system process“ (red), „response to external stimulus“ (blue) and „response to chemical“ (green) (figure 2) were highlighted in the respective colour. Top five key hub genes were highlighted with black rectangles. *Abbreviations*: Adj. = adjusted, BAL = bronchoalveolar lavage, Log2(FC) = Log2 fold change, LPS = lipopolysaccharide. Abbreviations of proteins are explained in supplementary table 1

In addition, a STRING protein-network analysis was performed to investigate the interactions among the significantly upregulated proteins identified in the LPS challenge model. The resulting network revealed different protein clusters of e.g., chemokines, matrix metalloproteinases or proteins that are implicated in immunoproteasome assembly (Fig. 3). The five key hub proteins within the network were IGHV3-43D (22 nodes), CD44 (16 nodes), EGFR (16 nodes), SYK (16 nodes) and FN1 (15 nodes), which all exhibited high connectivity to other proteins.

Chen et al. has published a systematic review summarizing current blood biomarkers of exacerbations in COPD^12^. In Table 2 of this review, 24 different biomarkers of studies with receiver operating characteristics (ROC) area-under-the curve (AUC) statistics are listed that were found to be elevated in AECOPD. We have checked whether these proteins were also increased in BAL following LPS challenge within our data set. Three (procalcitonin, RBP4, serum Amyloid-A 4) out of the 24 proteins were not measured using the SomaLogic SomaScan assay, and four biomarkers (BNP, CCL18, IFN-γ, IL-5) showed values below LLOD and were therefore not evaluable. From the remaining 17 proteins, 16 were significantly upregulated in BAL following LPS challenge compared to saline control (Table 2). The AECOPD biomarker sTREM-1 did not show changes in BAL collected after LPS challenge.

The concentrations of IL-6, IL-8 and MPO measured by the SomaLogic SomaScan assay highly correlated with previously published cytokine levels of the same study obtained by using the Meso Scale Discovery assay (supplementary Fig. 4a-c)^7^. Despite this high correlation, the SomaScan assay showed an overestimation of IL-6, IL-8 and MPO at lower concentration levels (supplementary Fig. 5a-c). No correlation between these two systems was observed for SP-D (supplementary Figs. 4 and 5d).

**Table 1:** Top 30 differentially expressed proteins (adj. p-value ≤ 0.05, |Log2FC| ≥ 0.58) in BAL following LPS challenge compared with saline challenge.

Protein name (Entrez gene symbol)	SomaScan sequence ID*	Log2FC	Adj. *p*-value
APOL1	11510-31	6.15	0.026
SAA4	15516-12	5.63	0.003
APOL1	11510-51	5.54	0.028
LILRA3	6391-52	4.98	0.007
CD5L	3293-2	4.41	0.004
VNN2	15480-2	4.28	0.002
APOA1	2750-3	4.26	0.002
CAMP	15481-45	4.18	0.003
ITIH4	4811-33	4.03	0.007
F11	2190-55	3.94	0.005
CNDP1	5456-59	3.86	0.012
HPX	2768-56	3.76	0.006
PLG	4151-6	3.71	0.003
MMP8	2954-56	3.69	0.002
CSF3	4840-73	3.68	0.002
IL6	4673-13	3.67	0.004
KNG1	7784-1	3.65	0.002
HGFAC	3617-80	3.63	0.002
SERPINA5	3389-7	3.62	0.002
PGLYRP1	3329-14	3.60	0.002
S100A8	25216-8	3.58	0.002
APCS	2474-54	3.57	0.006
BCHE	15514-26	3.57	0.003
SERPINB9	21221-67	3.56	0.008
TNFRSF1B	3152-57	3.56	0.003
C3	5803-24	3.53	0.005
MPO	2580-83	3.52	0.007
CPN2	6415-90	3.51	0.003
CD177	13116-25	3.46	0.003
F13A1|F13B	16927-9	3.46	0.004

*Interested scientists are encouraged to track additional information about the different SomaScan Sequence IDs by creating an account on the SomaLogic homepage (https://somalogic.com/).

**Table 2:** Expression of AECOPD biomarkers, according to table 2 in Chen et al.^12^, in BAL following LPS challenge compared with saline control challenge. Abbreviations: Adj. = adjusted, ID = identification, LLOD = lower limit of detection, Log2FC = Log2 fold change.

Biomarker according to Table 2 in Chen et al. [12]	SomaScan sequence ID	Log2FC	Adj. *p*-value
ADIPOQ / ARCP-30	3554-24	3.34	0.002
BNP	16751-15	LLOD	LLOD
CCL11 / Eotaxin	5301-7	2.03	0.003
CCL17	3519-3	2.04	0.008
CCL18 / PARC	3044-3	LLOD	LLOD
CCL23 / MPIF-1	3028-36	1.77	0.008
CCL23 / MPIF-1	2913-1	1.18	0.002
CRP	4337-49	2.44	0.009
CXCL10 / IP-10	4141-79	2.29	0.011
CXCL11	3038-9	2.63	0.007
CXCL8 / IL-8	3447-64	1.38	0.009
FGA|FGB|FGG / Fibrinogen	4907-56	2.70	0.008
ICAM1	4342-10	0.96	0.006
IFNγ	15346-31	LLOD	LLOD
IL-5	3741-4	LLOD	LLOD
IL-6	4673-13	3.67	0.004
MMP9	2579-17	2.07	0.007
PLAUR / suPAR	2652-15	1.31	0.002
Procalcitonin	no data	no data	no data
RBP4	no data	no data	no data
Serum Amyloid-A	no data	no data	no data
SFTPD / SP-D	4414-69	2.02	0.002
sTREM-1	9266-1	0.27	0.202
TNFRSF1A / TNFR1	2654-19	1.48	0.002
TNFRSF1B / TNFR2	3152-57	3.56	0.003
TNFRSF1B / TNFR2	8368 − 102	2.82	0.002

## Discussion

In this study, we performed a comprehensive proteomic analysis of BAL fluid following segmental LPS challenge in healthy participants, revealing significant insights into the inflammatory response induced by endotoxin exposure.

As expected, principal component analysis and hierarchical clustering allowed differentiation of BAL following LPS challenge from pre-challenge and post-saline challenge BAL, reinforcing the specificity and effectiveness of the LPS model in inducing localized inflammation in the human lung. Baseline BAL and BAL collected after saline control challenge clustered into a single group, which highlights the fact that the bronchoscopic procedure itself did not elicit an inflammatory response within the challenged lung segments. As expected based on the highly increased cell numbers and related changes within the transcriptomic profile in response to LPS^7,10^, endotoxin challenge resulted in upregulation of 40% of the analyzed proteins in BAL. Since primarily immune response-relevant proteins were selected for analysis, this high percentage of upregulated proteins was expected. Although two of the ten participants (S7 and S10) showed differences in the intensity of their immune response to LPS challenge, the overall protein expression patterns were consistent across all patients. As a result, hierarchical cluster analysis of the top 100 differentially expressed proteins did not identify distinct responder groups. Among the upregulated proteins were e.g., the cytokines IL-6 and IL-8, which play a crucial role in the immune response. Both cytokines were reported to be potential serum biomarkers for describing the severity and course of COPD exacerbations, and for predicting which patients are susceptible for future exacerbations^22–24^. Other significant upregulated proteins within the LPS challenge model were MPO and LTB4. Levels of both proteins were found to be increased in sputum of patients with bacterial versus non-bacterial AECOPD^25^ and therefore are potential biomarkers to guide antibiotic treatment during exacerbations. However, several proteins were below LLOD, which would have been expected to be increased after challenge with LPS (e.g., IL-2, IL-4, IL-13, IL-23, TSLP, IFN- γ, IL-1β, MCP-2, TNF-α, MIP-1α, MMP-3, MMP-8, WNT16, WNT5B, WNT7a). This likely reflects either an absence of these proteins in the samples or concentrations below the assay’s detection limit. It should be noted that the analyzed BAL samples were collected 24 h post LPS challenge, a timepoint where the kinetic peak of cytokine secretion is known to be almost back to baseline for many proinflammatory proteins^26^.

As there were too many genes to look individually at, gene set enrichment analysis (GSEA) analysis was performed. GSEA with the significantly upregulated proteins (adj. p-value ≤ 0.05, Log2FC ≥ 0.58) following LPS challenge revealed general pro-inflammatory processes, including the three top hits “*immune system process*”, “*response to external stimulus*”, and “*response to chemical*”. Additional upregulated processes were related to e.g., apoptosis and cell migration. All identified processes are crucial for inflammatory immune responses and align with the known pathophysiology of endotoxin-induced inflammation, further validating the model. The STRING protein-network analysis revealed key clusters, thereby providing a deeper insight into the complex interactions among the upregulated proteins in BAL following LPS challenge compared with saline. As expected, different chemokines (e.g., CCL2, CXCL8, CXCL10, CXCL11) were upregulated in response to LPS, which are known to play a major role in the recruitment of inflammatory cells, particularly neutrophil granulocytes, monocytes and T helper 1 cells^27^. Disbalance of the CCL2-CCR2, CXCL8-CXCR1/2, and CXCL9/10/11-CXCR3 axes play a role in the pathophysiology of COPD, both at a stable state and during exacerbations^28^. Another cluster consisted of different metalloproteinases such as MMP1, MMP2, MMP7, MMP9 and MMP10, which are critical for tissue homeostasis and development^29^. Disbalance of these proteolytic enzymes may contribute to airway remodeling and emphysema, which are major pathological features of COPD^30^. In addition to metalloproteinases, several other proteases such as neutrophil elastase (NE), cysteine proteases (CASP), or proteinase 3 (PR3) play key roles in COPD exacerbations by e.g., contributing to alveolar tissue destruction or regulating apoptosis^31–33^. PR3 was not included in the analysis panel. Signals for CASP8 and CASP10 were below LLOD, while CASP3 was significantly upregulated in BAL after LPS compared to saline challenge. As expected, values for neutrophil elastase (NE) were almost seven times higher in BAL after LPS compared to saline challenge (Saline: 194613.49 ± 81833.29, LPS: 1311714.93 ± 619835.68). Unfortunately, all signals in both groups were high above the upper limit of quantification (set to ≥ 100,000 as described in the method section) and therefore not evaluable. Furthermore, proteins that are related to immunoproteasome assembly (e.g., PSMB9, PSMB10, PSME1, PSME2^34,35^ build another cluster within the STRING protein-network. As the immunoproteasome improves major histocompatibility complex class I (MHC I)–mediated antigen presentation to defend against infections, impairment of the immunoproteasome function by e.g., cigarette smoke could lead to prolonged infections and exacerbations in COPD and idiopathic pulmonary fibrosis (IPF)^36^. Moreover, the key hub network-proteins IGHV3-43D (variations in the IGH locus impact the antibody repertoire^37^, CD44 (loss disrupts lung lipid surfactant homeostasis and exacerbates lung inflammation^38^, EGFR (major role in regulation of cell proliferation^39^, SYK (critical for signal transduction and antiviral responses^40^, and FN1 (involved in cell adhesion/motility^41^ are also known to have central roles in modulation of immune responses. In summary, GSEA- and protein-network analysis provided a deeper insight into ongoing mechanisms and pathways during the LPS-induced inflammatory response. As shown by the elaborated examples of chemokines, metalloproteinases, and immunoproteasome assembly, the human segmental LPS challenge model reflects some aspects of COPD and AECOPD. Targeting these pathways could be a possible intervention strategy for the treatment of COPD and AECOPD^28,42^. Furthermore, segmental LPS challenge could be a suitable model for respective first-in-human drug efficacy studies. The LPS challenge model has already been used in several clinical trials to investigate the anti-inflammatory effects of new investigational drugs. For example, treatment with the phosphodiesterase 4 inhibitor roflumilast^43^, the IL-1 receptor antagonist anakinra^44^ or the CXCR2 antagonist AZD8309^45^ have all attenuated pulmonary inflammation after segmental or inhalative LPS challenge due to decreased cell influx of in particular neutrophils, which are as already mentioned the predominant cell type in the airways of patients with COPD and AECOPD.

Chen et al. has published a list of biomarkers with ROC analysis that were found to be elevated in blood of patients with AECOPD^12^. We examined whether these proteins were also elevated in BAL following LPS challenge within our dataset. Unfortunately, there is no collection of relevant biomarkers for AECOPD in BAL so far, which would have been more suitable to identify proteomic similarities between AECOPD and the LPS challenge model. However, comparison of blood biomarkers with BAL collected within the LPS challenge model may help to identify matrix-independent biomarkers. 16 out of 17 evaluable biomarkers were elevated in both blood of patients with AECOPD as well as in BAL following LPS challenge, while only one AECOPD biomarker (sTREM-1) did not show differences within the LPS challenge model. In the literature, the expression of sTREM-1 in BAL as a biomarker for infectious pneumonia is controversially discussed, with studies reporting inconsistent results regarding its diagnostic utility^46,47^. In our study, levels of sTREM-1 did not differ significantly between BAL samples from LPS- and saline-challenged lung segments, which supports the assumption that sTREM-1 is unlikely to serve as a reliable BAL marker for distinguishing between bacterial and non-bacterial causes of inflammation. The high number of matching proteins further underlines the similarities between AECOPD and the segmental LPS challenge model. Therefore, the LPS challenge model could be suitable for studying inflammatory processes that are related to AECOPD as well as for first-in-human drug efficacy studies for the treatment of acute exacerbations. For each of the biomarkers elaborated by Chen et al., there is a large body of literature related to AECOPD. Most of the listed AECOPD biomarkers were chemokines (CXCL8/10/11 and CCL11/17/18/23), which, as already mentioned above, play a key role in the pathophysiology of COPD and exacerbations thereof^12,28^. In line, CXCL8 (synonym: IL-8), the most potent human neutrophil-attracting chemokine, was found to be elevated in sputum and BAL of patients with AECOPD^48,49^. Another AECOPD biomarker is CRP. While healthy individuals and patients with stable COPD show CRP concentrations below 10 mg/mL, higher levels are associated with an increased hospitalization and mortality risk^50^. Furthermore, serum CRP levels are elevated during viral and bacterial AECOPD, usually higher in bacterial triggered exacerbations^50^, which may could be useful in the guidance of antibiotic therapy^51,52^. High levels of the AECOPD biomarker fibrinogen was shown to predict the rate and risk of exacerbations in COPD, but not mortality or treatment response in a clinical trial^53^. However, both CRP and fibrinogen are acute phase reactants, which are not specific to an etiologic or biologic pathway, emphasizing the need of more specific biomarkers for the prognosis and diagnosis of AECOPD.

This study carries limitations. Although we found that LPS challenge reflects some aspects of AECOPD, the model induces transient airway inflammation and therefore does only partially reflect chronic inflammatory conditions as they are found in patients with stable COPD. For this reason, it is essential to perform comprehensive analysis of the transcriptomic and proteomic profile of the LPS challenge model in order to elaborate which features of patients with respiratory diseases are reflected in this model at the cellular and molecular level. Furthermore, we have analysed the proteomic profile of healthy non-smoking participants in this study. Tobacco smoke, which contains LPS^54,55^, is the major cause of COPD^1^. Therefore, smokers with chronic exposure to cigarette smoke may have been closer to the COPD phenotype than non-smoking participants. However, the inflammatory response to LPS challenge is similar or only slightly increased (e.g., IL-1β) in smoking compared to non-smoking participants^56^. Moreover, it should be mentioned that we have used the SomaScan assay platform in this study. To avoid interference with the assay system due to excessively high protein concentrations, we followed the manufacturer’s advice to standardize the total protein concentrations of all samples to 50 µg/mL. Total protein concentrations in BAL were 72.59 ± 20.56 µg/mL at baseline, 70.63 ± 17.60 µg/mL after saline, and 273.55 ± 114.87 µg/mL after LPS challenge. However, it cannot be ruled out that the dilution of particular BAL samples collected after LPS challenge led to signals below LLOD for some AECOPD relevant proteins. While concentrations of IL-6, IL-8 and MPO measured by the SomaLogic SomaScan assay highly correlated with previously published data obtained by using the Meso Scale Discovery (MSD) assay, levels of SP-D did not correlate between these two assay systems. Since SP-D is susceptible for conformational changes, in particular under inflammatory conditions^57^, and it is not known which exact epitope of SP-D is recognized by the respective antibody (MSD) or aptamer (SomaScan), it remains unclear whether the lack of correlation could be due to the molecule itself or due to method-specific reasons. In the SomaLogic SomaScan assay up to 11,000 proteins can be measured simultaneously within a samples^58^. This enables a comprehensive multi-proteomic analysis, which cannot be realized by conventional enzyme-linked immunosorbent assays, thereby opening new possibilities for future research and drug target screening.

## Conclusions

This study provides a very comprehensive analysis of the proteomic profile in BAL collected following LPS challenge. The identification of differentially expressed proteins, and the GSEA- as well as protein-network analysis allowed a deeper insight about ongoing mechanisms during LPS-induced inflammation. In summary, our data expanded the knowledge about the human segmental LPS challenge model and its similarities with infection-triggered respiratory inflammations such as AECOPD in particular.

## Supplementary Information

Below is the link to the electronic supplementary material.

Supplementary Material 1

Supplementary Material 2

Supplementary Material 3

Supplementary Material 4

Supplementary Material 5

## Data Availability

The datasets generated during and/or analyzed during the current study are available from the corresponding author JMH on reasonable request.

## Abbreviations

Adj.: Adjusted
BAL: Bronchoalveolar lavage
BMI: Body mass index
COPD: Chronic obstructive pulmonary disease
DPBS: Dulbecco’s phosphate-buffered saline
EU: Endotoxin units
FEV_1_: Forced expiratory volume in 1 s
FVC: Forced vital capacity
IL: Interleukin
Log2FC: Log 2-fold change
LPS: Lipopolysaccharide
N: Number of participants
PCA: Principal component analysis

## References

[1] Global Initiative for Chronic Obstructive Lung Disease. Global Strategy for the Diagnosis, Management, and Prevention of Chronic Obstructive Pulmonary Disease (2024 Report). Available at https://goldcopd.org/2024-gold-report/ (accessed 15 Jul 2024).

[2] Leist, M. & Hartung, T. Inflammatory findings on species extrapolations: humans are definitely 70-kg mice. *Arch. Toxicol.* **87**, 563–567. 10.1007/s00204-013-1038-0 (2013). 23503654 10.1007/s00204-013-1038-0 PMC3604596

[3] Mak, I. W. Y., Evaniew, N. & Ghert, M. Lost in translation: animal models and clinical trials in cancer treatment. *Am. J. Transl Res.* **6**, 114–118 (2014). 24489990 PMC3902221

[4] Gauvreau, G. M. & Evans, M. Y. Allergen inhalation challenge: a human model of asthma exacerbation. *Contrib. Microbiol.* **14**, 21–32. 10.1159/000107052 (2007). 17684330 10.1159/000107052

[5] Choy, R. K. M. et al. Controlled human infection models to accelerate vaccine development. *Clin. Microbiol. Rev.* **35**, e0000821. 10.1128/cmr (2022). 35862754 10.1128/cmr.00008-21 PMC9491212

[6] Brooks, D. et al. Human lipopolysaccharide models provide mechanistic and therapeutic insights into systemic and pulmonary inflammation. *Eur. Respir J.* **56**10.1183/13993003.01298-2019 (2020). 10.1183/13993003.01298-2019 32299854

[7] Holz, O., Müller, M., Carstensen, S., Olin, A-C. & Hohlfeld, J. M. Inflammatory cytokines can be monitored in exhaled breath particles following segmental and inhalation endotoxin challenge in healthy volunteers. *Sci. Rep.* **12**, 5620. 10.1038/s41598-022-09399-z (2022). 35379863 10.1038/s41598-022-09399-z PMC8979977

[8] Kharitonov, S. A. & Sjöbring, U. Lipopolysaccharide challenge of humans as a model for chronic obstructive lung disease exacerbations. *Contrib. Microbiol.* **14**, 83–100. 10.1159/000107056 (2007). 17684334 10.1159/000107056

[9] Korsgren, M. et al. Inhalation of LPS induces inflammatory airway responses mimicking characteristics of chronic obstructive pulmonary disease. *Clin. Physiol. Funct. Imaging*. **32**, 71–79. 10.1111/j.1475-097X.2011.01058.x (2012). 22152082 10.1111/j.1475-097X.2011.01058.x

[10] Gress, C. et al. Transcriptomic characterization of the human segmental endotoxin challenge model. *Sci. Rep.* **14**, 1721. 10.1038/s41598-024-51547-0 (2024). 38242945 10.1038/s41598-024-51547-0 PMC10798985

[11] Holz, O. et al. Changes of breath volatile organic compounds in healthy volunteers following segmental and inhalation endotoxin challenge. *J. Breath. Res.* **16**10.1088/1752-7163/ac6359 (2022). 10.1088/1752-7163/ac6359 35366648

[12] Chen, Y-W-R., Leung, J. M. & Sin, D. D. A systematic review of diagnostic biomarkers of COPD exacerbation. *PLoS One*. **11**, e0158843. 10.1371/journal.pone.0158843 (2016). 27434033 10.1371/journal.pone.0158843 PMC4951145

[13] Workshop summary and. Guidelines: investigative use of bronchoscopy, lavage, and bronchial biopsies in asthma and other airway diseases. *J. Allergy Clin. Immunol.* **88**, 808–814. 10.1016/0091-6749(91)90189-u (1991). 1955640 10.1016/0091-6749(91)90189-u

[14] Du Rand, I. A. et al. Summary of the British thoracic society guidelines for advanced diagnostic and therapeutic flexible bronchoscopy in adults. *Thorax* **66**, 1014–1015. 10.1136/thoraxjnl-2011-201052 (2011). 22003155 10.1136/thoraxjnl-2011-201052

[15] Gold, L. et al. Aptamer-based multiplexed proteomic technology for biomarker discovery. *Nat. Prec*. **5**, e15004. 10.1038/npre.2010.4538.1 (2010). 10.1371/journal.pone.0015004 PMC3000457 21165148

[16] Rohloff, J. C. et al. Nucleic acid ligands with Protein-like side chains: modified aptamers and their use as diagnostic and therapeutic agents. *Mol. Ther. Nucleic Acids*. **3**, e201. 10.1038/mtna.2014.49 (2014). 25291143 10.1038/mtna.2014.49 PMC4217074

[17] Candia, J. et al. Assessment of variability in the SOMAscan assay. *Sci. Rep.* **7**, 14248. 10.1038/s41598-017-14755-5 (2017). 29079756 10.1038/s41598-017-14755-5 PMC5660188

[18] Cheung, F. et al. Web tool for navigating and plotting somalogic ADAT files. *J. Open. Res. Softw.* **5**, 20. 10.5334/jors.166 (2017). 29951204 10.5334/jors.166 PMC6017986

[19] Raudvere, U. et al. g:Profiler: a web server for functional enrichment analysis and conversions of gene lists (2019 update). *Nucleic Acids Res.* **47** (198), W191–W. 10.1093/nar/gkz369 (2019). 31066453 10.1093/nar/gkz369 PMC6602461

[20] Szklarczyk, D. et al. The STRING database in 2023: protein-protein association networks and functional enrichment analyses for any sequenced genome of interest. *Nucleic Acids Res.* **51**, D638–D646. 10.1093/nar/gkac1000 (2023). 36370105 10.1093/nar/gkac1000 PMC9825434

[21] Metsalu, T. & Vilo, J. ClustVis: a web tool for visualizing clustering of multivariate data using principal component analysis and heatmap. *Nucleic Acids Res.* **43**, W566–W570. 10.1093/nar/gkv468 (2015). 25969447 10.1093/nar/gkv468 PMC4489295

[22] Huang, H. et al. Interleukin-6 is a strong predictor of the frequency of COPD exacerbation within 1 year. *Int. J. Chron. Obstruct Pulmon Dis.* **16**, 2945–2951. 10.2147/COPD.S332505 (2021). 34737559 10.2147/COPD.S332505 PMC8560075

[23] Hussein, F. G. M., Mohammed, R. S., Khattab, R. A. & Al-Sharawy, L. A. Serum interleukin-6 in chronic obstructive pulmonary disease patients and its relation to severity and acute exacerbation. *Egypt. J. Bronchol.* **16**10.1186/s43168-022-00115-z (2022).

[24] Zhang, J. & Bai, C. The significance of serum Interleukin-8 in acute exacerbations of chronic obstructive pulmonary disease. *Tanaffos* **17**, 13–21 (2018). 30116274 PMC6087525

[25] Bathoorn, E. et al. Change in inflammation in out-patient COPD patients from stable phase to a subsequent exacerbation. *Int. J. Chron. Obstruct Pulmon Dis.* **4**, 101–109. 10.2147/copd.s4854 (2009). 19436694 10.2147/copd.s4854 PMC2672798

[26] Holz, O. et al. Inter- and intrasubject variability of the inflammatory response to segmental endotoxin challenge in healthy volunteers. *Pulm Pharmacol. Ther.* **35**, 50–59. 10.1016/j.pupt.2015.10.011 (2015). 26545873 10.1016/j.pupt.2015.10.011

[27] Barnes, P. J. Immunology of asthma and chronic obstructive pulmonary disease. *Nat. Rev. Immunol.* **8**, 183–192. 10.1038/nri2254 (2008). 18274560 10.1038/nri2254

[28] Henrot, P., Prevel, R., Berger, P. & Dupin, I. Chemokines in COPD: from implication to therapeutic use. *Int. J. Mol. Sci.* **20**10.3390/ijms20112785 (2019). 10.3390/ijms20112785 PMC6600384 31174392

[29] Löffek, S., Schilling, O. & Franzke, C-W. Series matrix metalloproteinases in lung health and disease: biological role of matrix metalloproteinases: a critical balance. *Eur. Respir J.* **38**, 191–208. 10.1183/09031936.00146510 (2011). 21177845 10.1183/09031936.00146510

[30] Christopoulou, M-E., Papakonstantinou, E. & Stolz, D. Matrix metalloproteinases in chronic obstructive pulmonary disease. *Int. J. Mol. Sci.* **24**10.3390/ijms24043786 (2023). 10.3390/ijms24043786 PMC9966421 36835197

[31] Thulborn, S. J. et al. Neutrophil elastase as a biomarker for bacterial infection in COPD. *Respir Res.* **20**, 170. 10.1186/s12931-019-1145-4 (2019). 31362723 10.1186/s12931-019-1145-4 PMC6668103

[32] Sinden, N. J. & Stockley, R. A. Proteinase 3 activity in sputum from subjects with alpha-1-antitrypsin deficiency and COPD. *Eur. Respir J.* **41**, 1042–1050. 10.1183/09031936.00089712 (2013). 22936713 10.1183/09031936.00089712

[33] Pandey, K. C., De, S. & Mishra, P. K. Role of proteases in chronic obstructive pulmonary disease. *Front. Pharmacol.* **8**, 512. 10.3389/fphar.2017.00512 (2017). 28848433 10.3389/fphar.2017.00512 PMC5550664

[34] Watanabe, A., Yashiroda, H., Ishihara, S., Lo, M. & Murata, S. The molecular mechanisms governing the assembly of the Immuno- and thymoproteasomes in the presence of constitutive proteasomes. *Cells* **11**10.3390/cells11091580 (2022). 10.3390/cells11091580 PMC9105311 35563886

[35] GomesAV Genetics of proteasome diseases. *Scientifica (Cairo)*. **637629**10.1155/2013/637629 (2013). (2013). 10.1155/2013/637629 PMC3892944 24490108

[36] Kammerl, I. E. et al. Impairment of Immunoproteasome function by cigarette smoke and in chronic obstructive pulmonary disease. *Am. J. Respir Crit. Care Med.* **193**, 1230–1241. 10.1164/rccm.201506-1122OC (2016). 26756824 10.1164/rccm.201506-1122OC

[37] Rodriguez, O. L. et al. Genetic variation in the Immunoglobulin heavy chain locus shapes the human antibody repertoire. *Nat. Commun.* **14**, 4419. 10.1038/s41467-023-40070-x (2023). 37479682 10.1038/s41467-023-40070-x PMC10362067

[38] Dong, Y. et al. CD44 loss disrupts lung lipid surfactant homeostasis and exacerbates oxidized lipid-Induced lung inflammation. *Front. Immunol.* **11**, 29. 10.3389/fimmu.2020.00029 (2020). 32082314 10.3389/fimmu.2020.00029 PMC7002364

[39] Wee, P. & Wang, Z. Epidermal growth factor receptor cell proliferation signaling pathways. *Cancers (Basel)*. **9** (52). 10.3390/cancers9050052 (2017). 10.3390/cancers9050052 PMC5447962 28513565

[40] Liu, S. et al. Critical role of Syk-dependent STAT1 activation in innate antiviral immunity. *Cell. Rep.* **34**, 108627. 10.1016/j.celrep.2020.108627 (2021). 33472080 10.1016/j.celrep.2020.108627

[41] Zollinger, A. J. & Smith, M. L. Fibronectin, the extracellular glue. *Matrix Biol.* **60–61**, 27–37. 10.1016/j.matbio.2016.07.011 (2017). 27496349 10.1016/j.matbio.2016.07.011

[42] Barnes, P. J. & Stockley, R. A. COPD: current therapeutic interventions and future approaches. *Eur. Respir J.* **25**, 1084–1106. 10.1183/09031936.05.00139104 (2005). 15929966 10.1183/09031936.05.00139104

[43] Hohlfeld, J. M. et al. Roflumilast attenuates pulmonary inflammation upon segmental endotoxin challenge in healthy subjects: a randomized placebo-controlled trial. *Pulm Pharmacol. Ther.* **21**, 616–623. 10.1016/j.pupt.2008.02.002 (2008). 18374614 10.1016/j.pupt.2008.02.002

[44] Hernandez, M. L. et al. IL-1 receptor antagonist reduces endotoxin-induced airway inflammation in healthy volunteers. *J. Allergy Clin. Immunol.* **135**, 379–385. 10.1016/j.jaci.2014.07.039 (2015). 25195169 10.1016/j.jaci.2014.07.039 PMC4323893

[45] Leaker, B. R., Barnes, P. J. & O’Connor, B. Inhibition of LPS-induced airway neutrophilic inflammation in healthy volunteers with an oral CXCR2 antagonist. *Respir Res.* **14**, 137. 10.1186/1465-9921-14-137 (2013). 24341382 10.1186/1465-9921-14-137 PMC3867427

[46] Gibot, S. et al. Soluble triggering receptor expressed on myeloid cells and the diagnosis of pneumonia. *N Engl. J. Med.* **350**, 451–458. 10.1056/NEJMoa031544 (2004). 14749453 10.1056/NEJMoa031544

[47] Palazzo, S. J., Simpson, T. A., Simmons, J. M. & Schnapp, L. M. Soluble triggering receptor expressed on myeloid cells-1 (sTREM-1) as a diagnostic marker of ventilator-associated pneumonia. *Respir Care*. **57**, 2052–2058. 10.4187/respcare.01703 (2012). 22613763 10.4187/respcare.01703 PMC4432465

[48] Aaron, S. D. et al. Granulocyte inflammatory markers and airway infection during acute exacerbation of chronic obstructive pulmonary disease. *Am. J. Respir Crit. Care Med.* **163**, 349–355. 10.1164/ajrccm.163.2.2003122 (2001). 11179105 10.1164/ajrccm.163.2.2003122

[49] Tumkaya, M. et al. Relationship between airway colonization, inflammation and exacerbation frequency in COPD. *Respir Med.* **101**, 729–737. 10.1016/j.rmed.2006.08.020 (2007). 17002892 10.1016/j.rmed.2006.08.020

[50] Celli, B. R. et al. An updated definition and severity classification of chronic obstructive pulmonary disease exacerbations: the Rome proposal. *Am. J. Respir Crit. Care Med.* **204**, 1251–1258. 10.1164/rccm.202108-1819PP (2021). 34570991 10.1164/rccm.202108-1819PP

[51] Butler, C. C. et al. C-Reactive protein testing to guide antibiotic prescribing for COPD exacerbations. *N Engl. J. Med.* **381**, 111–120. 10.1056/NEJMoa1803185 (2019). 31291514 10.1056/NEJMoa1803185

[52] Miravitlles, M., Moragas, A., Hernández, S., Bayona, C. & Llor, C. Is it possible to identify exacerbations of mild to moderate COPD that do not require antibiotic treatment? *Chest* **144**, 1571–1577. 10.1378/chest.13 (2013). 23807094 10.1378/chest.13-0518

[53] Singh, D. et al. InforMing the pathway of COPD treatment (IMPACT) trial: fibrinogen levels predict risk of moderate or severe exacerbations. *Respir Res.* **22**, 130. 10.1186/s12931-021-01706-y (2021). 33910578 10.1186/s12931-021-01706-y PMC8080358

[54] Hasday, J. D., Bascom, R., Costa, J. J., Fitzgerald, T. & Dubin, W. Bacterial endotoxin is an active component of cigarette smoke. *Chest* **115**, 829–835. 10.1378/chest.115.3.829 (1999). 10084499 10.1378/chest.115.3.829

[55] Larsson, L., Pehrson, C., Dechen, T. & Crane-Godreau, M. Microbiological components in mainstream and sidestream cigarette smoke. *Tob Induc Dis* 10, 13; (2012). 10.1186/1617-9625-10-13 10.1186/1617-9625-10-13 PMC3444954 22898193

[56] Wesselius, L. J., Nelson, M. E., Bailey, K. & O’Brien-Ladner, A. R. Rapid lung cytokine accumulation and neutrophil recruitment after lipopolysaccharide inhalation by cigarette smokers and nonsmokers. *J. Lab. Clin. Med.* **129**, 106–114. 10.1016/s0022-2143(97)90167-0 (1997). 9011586 10.1016/s0022-2143(97)90167-0

[57] Atochina-Vasserman, E. N. et al. Segmental allergen challenge alters multimeric structure and function of surfactant protein D in humans. *Am. J. Respir Crit. Care Med.* **183**, 856–864. 10.1164/rccm.201004-0654OC (2011). 21131470 10.1164/rccm.201004-0654OC PMC3086753

[58] Candia, J. et al. Variability of 7K and 11K SomaScan plasma proteomics assays. *J. Proteome Res.* **23**, 5531–5539. 10.1021/acs.jproteome.4c00667 (2024). 39473295 10.1021/acs.jproteome.4c00667 PMC11629374

