# Supplementary material for: Proteomic profiling of bronchoalveolar lavage following human segmental endotoxin challenge—a potential exacerbation model
Source: Sci Rep. 2026 Feb 12;16:6145. doi: 10.1038/s41598-026-39528-x (PMC12902028; doi:10.1038/s41598-026-39528-x)
Supplement: Supplementary file 5 — Supplementary Material 5 [file 41598_2026_39528_MOESM5_ESM.docx]

# Supplementary figures

**Supplementary figure 1.** For analysis of the data obtained by the SomaLogic SomaScan assay an upper limit of detection (ULOD) was needed. As dilution linearity was given for values below < 100,000 RFU (a-d), but not for values above > 100,000 RFU (e-l), this value was defined as cutoff signal. Proteins with signal values above the ULOD were excluded from further analysis. Abbreviations: C3 = Complement C3, CFI = Complement factor I, CXCL1 = C-X-C Motif Chemokine Ligand 1, ENO1 = Alpha-enolase, IL = Interleukin, LCN2 = Lipocalin 2, LTF = Lactotransferrin, MPO = Myeloperoxidase, PPIA = Peptidyl-prolyl cis-trans isomerase A, RFU = Relative Fluorescence Unit.


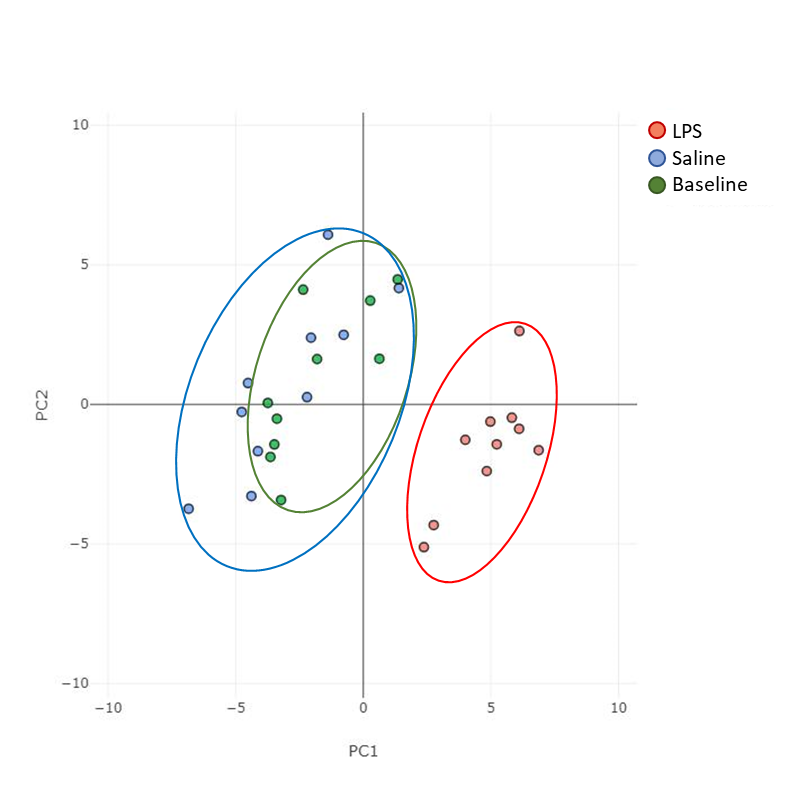


**Supplementary figure 2.** Principal component analysis of BAL collected in the LPS challenge model at pre-challenge baseline (green), and following LPS (red) or saline (blue) challenge. Abbreviations: BAL = bronchoalveolar lavage, LPS = lipopolysaccharide, PC = principal component.


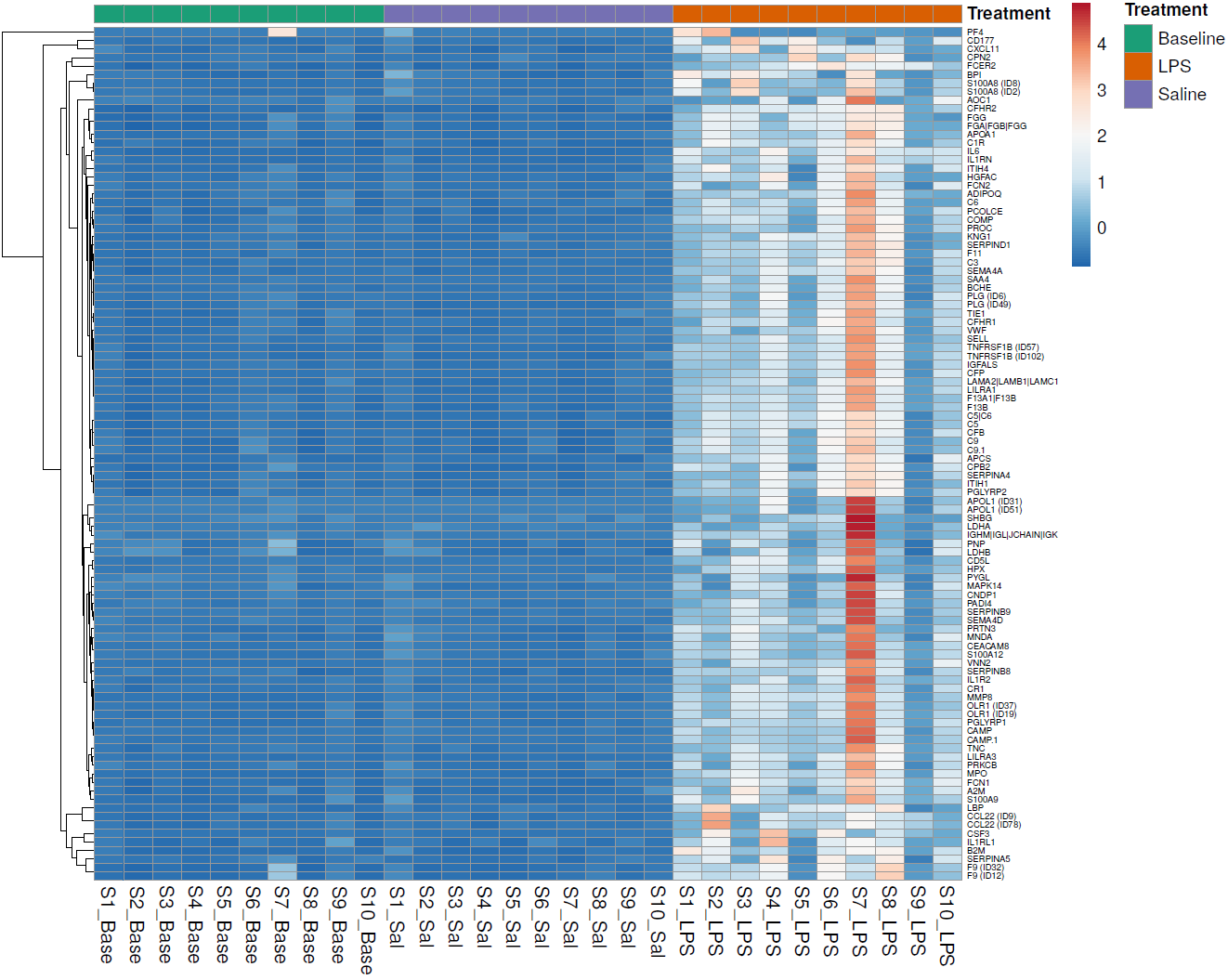


**Supplementary figure 3.** Hierarchical protein clustering of the top 100 differentially expressed proteins with the highest Log2FC after LPS compared to saline challenge. Treatment: BAL collected at baseline before challenge (green), post saline control (violet), and post LPS (orange) challenge. Abbreviations: BAL = bronchoalveolar lavage, Base = Baseline, LPS = lipopolysaccharide, PC = principal component, S = Subject, Sal = Saline. ID numbers in brackets represent the suffix of different SOMAmer reagents targeting the same protein.

**Supplementary figure 4.** Spearman correlation analysis of measurements for IL-6, IL-8, MPO and SP-D in BAL obtained by the SomaLogic SomaScan assay compared to previously published data obtained by using the Meso Scale Discovery assay [7]. Abbreviations: BAL = bronchoalveolar lavage, IL = interleukin, MPO = myeloperoxidase, MSD = Meso Scale Discovery, RFU = Relative Fluorescence Unit, SP-D = surfactant protein D.

**Supplementary figure 5.** Bland-Altman plot for measurements for IL-6, IL-8, MPO and SP-D in BAL obtained by the SomaLogic SomaScan assay compared to previously published data obtained by using the Meso Scale Discovery assay [7]. Abbreviations: BAL = bronchoalveolar lavage, IL = interleukin, MPO = myeloperoxidase, SP-D = surfactant protein D.
